# Kernel machine tests of association using extrinsic and intrinsic cluster evaluation metrics

**DOI:** 10.1371/journal.pcbi.1012524

**Published:** 2024-11-11

**Authors:** Alexandria M. Jensen, Peter DeWitt, Brianne M. Bettcher, Julia Wrobel, Katerina Kechris, Debashis Ghosh

**Affiliations:** 1 Quantitative Sciences Unit, Stanford School of Medicine, Palo Alto, California, United States of America; 2 Department of Biomedical Informatics, University of Colorado School of Medicine, Aurora, Colorado, United States of America; 3 Behavioral Neurology Section, Department of Neurology, University of Colorado Alzheimer’s and Cognitition Center, Aurora, Colorado, United States of America; 4 Department of Biostatistics and Informatics, Colorado School of Public Health, Aurora, Colorado, United States of America; Brown University, UNITED STATES OF AMERICA

## Abstract

Modeling the network topology of the human brain within the mesoscale has become an increasing focus within the neuroscientific community due to its variation across diverse cognitive processes, in the presence of neuropsychiatric disease or injury, and over the lifespan. Much research has been done on the creation of algorithms to detect these mesoscopic structures, called communities or modules, but less has been done to conduct inference on these structures. The literature on analysis of these community detection algorithms has focused on comparing them within the same subject. These approaches, however, either do not accomodate a more general association between community structure and an outcome or cannot accommodate additional covariates that may confound the association of interest. We propose a semiparametric kernel machine regression model for either a continuous or binary outcome, where covariate effects are modeled parametrically and brain connectivity measures are measured nonparametrically. By incorporating notions of similarity between network community structures into a kernel distance function, the high-dimensional feature space of brain networks, defined on input pairs, can be generalized to non-linear spaces, allowing for a wider class of distance-based algorithms. We evaluate our proposed methodology on both simulated and real datasets.

## Author summary

Parcellating the brain into clusters, which can be characterized as a way to describe the balance between dense relationships among areas highly engaged in the same processing tasks as well as sparser relationships between regions with different processing assignments, has been an area of recent focus within the neuroscientific community. While many algorithms exist to discover and characterize these clusters, there is a paucity of literature seeking to conduct inference on these parcellations between subjects. We have proposed a semiparametric kernel machine regression framework that can accommodate either a binary or continuous outcome, where brain connectivity measures are modeled nonparametrically and any additional covariates of interest (e.g., age, sex, etc.) are modeled parametrically. Evaluating our proposed methodology on both simulated and real datasets, we provide evidence of the robustness of this method within a single layer of clusters, thus showing the potential utility in the rapidly-changing field of network neuroscience.

## Introduction

In the years since Bullmore and Sporns’ landmark 2009 paper that first applied network analysis techniques to the question of brain connectivity [1], research in this field has exploded, due to the parallel maturations of the neuroimaging and network science fields [2]. Broadly, connectivity research is concerned with the anatomical links, statistical dependencies, or causal interactions between distinct units in a nervous system. While initial studies focused on local (i.e., groups of neurons) and global (i.e., the entire brain) characteristics [3, 4], the spatiotemporal patterns of connectivity can be seen as a hierarchy based on the ideas of segregation and integration [2]. These intermediate characteristics, known as the mesoscale, span the entire range of organization, from small, functionally specialized areas to larger regions for general brain function and cognition [5]. This organization displays what Herbert Simon deemed as a “nearly-decomposable system,” which is a system where elements have most of their interactions with a subset of elements close to them and much less interaction outside of this subset [6, 7]. Simon argued that a network with near-decomposability posed an evolutionary advantage as modules allow for a stable middle ground as complex systems emerge from more simplistic formations [7]. Similarly, many complex networks are also characterized by small-world topology, where the network is composed of dense clustering connections amongst neighboring nodes; this allows for short path lengths between any two nodes and the minimization of long-range connections [8]. Like most man-made and natural information processing systems, the modular (or community) organization of the brain, and within it the characterizations of near-decomposability and small-world topology, is thought to be a method of minimizing energy consumption in the development and maintenance of connections [9]. The composition of these modules underpins the communication patterns of the brain and promotes a well-balanced and efficient mechanism for integration and segregation [3, 10]. The architecture of mesoscale brain networks has been shown to vary over the lifespan [11–14], in the presence of neuropsychiatric disease or injury [15–18], and across diverse cognitive processes [19].

There exist an assortment of methods and algorithms for detecting network communities. Fortunato [20] and later with Newman [21] provide excellent summaries of the various approaches to community detection, as well as Yang, Algesheimer, and Tessone in 2016 [22] and Mukerjee in 2021 [23]. A recent interest in the literature has been topological data analysis, which uses persistent homology to represent the shape of and cluster data [24]. The utility of topological data analysis in neuroscience is beginning to be explored [25, 26], but, much like any other community detection algorithm, is not without its pitfalls and challenges. In the context of brain connectivity networks, once a community detection algorithm has been run on the data, understanding whether the pattern of assigning nodes to communities varies based on some outcome of interest is the next logical step in the analytic pipeline. However, much of the neuroscience literature on analysis of these community detection algorithms has focused on comparing them within the same subject. Taya et al. used a non-parametric permutation test based on the normalized mutual information to determine which algorithm or atlas provided the best representative structure [27]. Within multilayer or mulitplex networks, measuring layer similarity has been explored by [28] as well as [29]. Onnela et al. compared networks by creating profiles of summary statistics that characterized the community structure of each network at different mesoscopic scales [30]. Bródka et al. quantified relative differences by comparing aggregations of layer property vectors, with a distance between observed distributions of these properties based on histograms [31]. Betzel et al. used a similar resolution parameter approach, but then quantified the average dissimilarity of an individual’s community assignments across layers to all other subjects, creating entropy vectors that were then used as an input for a principal component analysis [5]. Lohse et al. created a summary of graph summary measures (bipartivity, community laterality, community radius, number of singletons) to create mesoscopic response functions to describe multi-resolution topological structure in various networks [32]. Finally, Alexander-Bloch et al. compared summary measures of community structures (e.g., normalized mutual information) between groups using a permutation test and then used Pearson’s phi to assess regionally-specific differences in community structure [33]. These approaches, however, may not be flexible enough to model the linear and nonlinear effects of potential confounders and community structures on an outcome of interest.

One way to accomplish this is through the use of kernels, which are weighting functions used to estimate the conditional expectation of a random variable [34]. Kernels are a powerful tool within machine learning for multidimensional data, with uses ranging from support vector machines [35] to spatial smoothing for functional MRI analyses [36]. Unlike spline-based models, which require the specification of a smoothness condition of an unknown function, the kernel function in a kernel machine method implicitly determines the smoothness property of the unknown function [34]. When working in terms of a kernel function, one can avoid the explicit introduction of a feature vector, therefore allowing for implicit use of feature spaces of high dimensionality, something linear statistcal models lack. Kernel-based methods do not make assumptions on the functional form of the association, assuming a more general relationship based on a Hilbert space of functions.

This study proposes a semiparametric kernel machine regression model for covariate and brain connectivity measures on either a continuous or binary outcome, where covariate effects are modeled parametrically and brain connectivity measures are measured nonparametrically. As defining a measure of network similarity is a non-trivial task, our approach has the flexibility to incorporate either extrinsic or intrinsic cluster evaluation metrics [37]. By incorporating these notions of similarity into a kernel distance function, the high-dimensional feature space of brain networks, defined on input pairs, can be generalized to non-linear spaces; this allows for a wider class of distance-based algorithms, to be used for modeling the similarity between two network community structures.

The remainder of this paper is organized as follows. A description of the cluster evaluation metrics, the general framework for distance-based kernels, and the kernel machine tests of association can be found in the **Materials and Methods** section. In **Results**, we apply these methods under a variety of simulation paradigms using a novel simulation technique and to a subsampled dataset from the publicly available, pre-processed Autism Brain Imaging Data Exchange (ABIDE) study. Finally, we conclude the paper with a discussion and proposal of future directions. All code has been made available in the form of an R package published on CRAN, CommKern (https://cran.r-project.org/web/packages/CommKern/index.html).

## Materials and methods

### Cluster evaluation metrics

Within community detection, the methods for evaluating the performance of a particular algorithm can be classified as either (1) extrinsic, requiring ground truth labels or (2) intrinsic, not requiring ground truth labels. Focusing on extrinsic evaluation, metrics can be further groups into four families: (1) metrics based on counting pairs; (2) based on set matching; (3) based on entropy; and (4) based on edit distance. All external clustering measures rely on a *r* × *k* contingency matrix ***N*** that is induced by a system-clustering C and a ground truth partitioning T, where the rows represent the system-clusters $C_{i}\in C$ for *i* = 1, …, *r* and the columns represent the ground truth partitions $T_{j}\in T$ for *j* = 1, …, *k*. A cell at the intersection of row *i* and column *j* contains the count *n*_*ij*_ of points that are common to cluster $C_{i}$ and ground truth partition $T_{j}$:

$$\begin{array}{c} N(i,j)=n_{ij}=\left|C_{i}\cap T_{j}\right| \end{array}$$ (1)

[28]. In many cases, however, a ground truth labelling does not exist, such as in the case of brain connectivity. To overcome this issue in the context of between layer communities, Ghawi and Pfeffer proposed a two-way matching be performed, where the matching is performed twice, each time with one of the layer’s clusterings playing the role of “ground truth” and the results combined using a harmonic mean to produce a final matching measure [28]. We have extended this logic to comparing clusterings between two graphs, $G_{i}$ and $G_{j}$. This approach overcomes the lack of a ground truth clustering commonly seen in real-world applications while preserving the interpretation of such extrinsic clustering metrics.

There are several options for external cluster evaluation metrics. For all, assume Ω = {*ω*_1_, *ω*_2_, …, *ω*_*k*_} and $C=\{c_{1},c_{2},\ldots ,c_{j}\}$ are two sets of clusters.

**Definition 1. *Purity***: *a measure that quantifies the extent to which a cluster c*_*i*_ *contains elements from only one partition, or measures how “pure” a cluster is* [28]. *The formal definition of purity for a clustering* C *is given by*

$$\begin{array}{c} purity(\Omega ,C)=\frac{1}{N}\sum_{k}\;\underset{j}{\text{max}}\;\left|\omega_{k}\cap c_{j}\right|=\frac{1}{N}\sum_{i=1}^{r}\;\overset{k}{\underset{j=1}{\text{max}}}\;\{n_{ij}\} \end{array}$$ (2)

[28]. *Purity is bounded in* [0, 1] *but is not symmetric due to the need to have a ground truth labeling. To get around this issue, the harmonic mean of the purity between two clusterings is taken, where one partition is taken to be the ground truth labeling, then the other*.

**Definition 2. *Normalized Mutual Information (NMI)***: *a measure that quantifies the “amount of information” obtained about one random variable by observing the other and is linked to the concept of entropy, which attempts to characterize the “unpredictability” of a random variable. Mutual information is both non-negative and symmetric. Normalized variants of mutual information allow for comparisons between different clusterings that have different numbers of clusters (but not a different number of nodes, meaning the underlying networks must be of the same size). The formal definition of the normalized mutual information is given by*

$$\begin{array}{c} NMI(\Omega ,C)=\frac{I(\Omega ,C)}{[H(\Omega )+H(C)]/2}. \end{array}$$ (3)

I(Ω,C) *is the mutual information, defined as*

$$\begin{array}{c} I(\Omega ,C)=\sum_{k}\sum_{j}P(\omega_{k}\cap c_{j})\;\text{ln}\;\frac{P(\omega_{k}\cap c_{j})}{P(\omega_{k})\;P(c_{j})} \end{array}$$ (4)

*where P*(*ω*_*k*_), *P*(*c*_*j*_), *and P*(*ω*_*k*_ ∩ *c*_*j*_) *are the probabilities of a node being in cluster ω*_*k*_, *cluster c*_*j*_, *and in the intersection of ω*_*k*_ *and c*_*j*_, *respectively. H is the entropy, defined as*

$$\begin{array}{c} H(\Omega )=-\sum_{k}P(\omega_{k})\;\text{ln}\;P(\omega_{k}) \end{array}$$ (5)

[38]. I(Ω,C) *in* Eq (4) *measures the amount of information by which our knowledge of the one clustering increases when we know the other. The normalization by the denominator* [H(Ω)+H(C)]/2 *in* Eq 3 *ensures that the NMI is always in* [0, 1].

**Definition 3. *Adjusted Rand Index (ARI)***: *An alternative to an information-theoretic interpretation of clustering evaluation (e.g., NMI, purity) is to view clusterings as a series of decisions, one for each of the N*(*N* − 1)/2 *pairs of nodes in the network. If we define the following*:

- *a: the number of pairs of nodes in a network that are in the same cluster in* Ω *and in the same cluster in* C
- *b: the number of pairs of nodes in a network that are in different clusters in* Ω *and in different clusters in* C
- *c: the number of pairs of nodes in a network that are in the same cluster in* Ω *and in different clusters in* C
- *d: the number of pairs of nodes in a network that are in different clusters in* Ω *and the same cluster in* C,

*then the Rand Index* [39] *is*

$$\begin{array}{c} RI=\frac{a+b}{a+b+c+d}=\frac{a+b}{N(N+1)/2}. \end{array}$$ (6)

*Intuitively, a* + *b can be considered the number of agreements between* Ω *and* C, *while c* + *d the number of disagreements between* Ω *and* C. *The adusted Rand Index (ARI) is the corrected-for-chance version of the Rand Index, which establishes a baseline by using the expected similarity of all pairwise comparisons between clusterings specified by a random model*.

ARI=∑ij(nij2)-[∑i(ai2)∑j(bj2)]/(n2)12[∑i(ai2)+∑j(bj2)]-[∑i(ai2)∑j(bj2)]/(n2), (7)

*where for the contingency table detailed in* Eq (1), *n*_*ij*_ *is the diagonal (when i* = *j*), *a*_*i*_ *are the row sums, and b*_*j*_ *are the column sums. Both the RI and ARI are symmetric, but while the RI can only yield values in* [0, 1], *the ARI can yield negative values if the index is less than the expected index*.

Each of these extrinsic cluster evaluation metrics can be commonly found within the literature but have their drawbacks. Purity generally increases as the number of clusters increases, thus it cannot be used as a tradeoff between the number of clusters and clustering quality. NMI has the potential to be biased because the symmetic normalization can introduce spurious dependence on the labeling. Finally, ARI assumes a hypergeometric distribution, which is not always appropriate if the clusterings are dependent and can be biased in the presence of heavy cluster size imbalance.

Rather than use extrinsic metrics, which rely either on a ground truth labeling or on a harmonic mean between two clusterings, intrinsic evaluation metrics do not rely on the existence of a ground truth. One of the most commonly used intrinsic metrics is the optimized value of the modularity function; this is based on the fact that networks with very similar community structure should have very similar modularity values. However, not all community detection algorithms find optimal partitions through use of a modularity function; for example, the spinglass algorithm, first proposed by [40], is optimized by finding the ground state of a spinglass model using a Potts Hamiltonian. Use of the optimized modularity or Hamiltonian value from a community detection algorithm as an intrinsic evaluation metric serve the same purpose, as was first proposed by [30]. In it, they created mesoscopic response functions of the spinglass Hamiltonian across a range of resolution parameters and looked at the distance between the two functions in a more qualitative manner [30]. Because the optimized Hamiltonian or modularity values are not bounded in the same manner as the extrinsic cluster evaluation metrics, a p-norm of the differences between these values for two clusterings was used such that

$$\begin{array}{c} d(H_{\Omega },H_{C})={\left\|H_{\Omega }-H_{C}\right\|}_{p}\;\text{for}\;1\leq p<\infty . \end{array}$$ (8)

It should be noted, however, that Emmons et al. argues that the lack of a rigorous definition of a community, even within domain, “is the root of disrepancies amongst stand-alone quality metrics and information recovery metrics [41].” Therefore, it is left to the reader to determine which cluster evaluation metric best suits their dataset and carefully interpret the results of such metrics through this lens.

### Kernels

Most approaches used in the analysis of graph theoretic measures are based on linear methods, ranging from t-tests to analysis of variance (ANOVA) to multivariable regression. While these methods have thoroughly studied properties and simple mathematical forms, many times analysis on real data requires a nonlinear methodological approach. The brain is an especially complex system to model, whose dynamics are non-linear at multiple levels [42]. Kernels provide a computationally efficient and mathematically tractable method of extending linear methods into nonlinear ones. This work will provide a brief overview of kernels; for a more comprehensive examination, please refer to [43–45].

Broadly speaking, rather than assuming a linear functional relationship, kernel-based methods assume a more general (i.e., non-linear) relationship. A kernel is a function that takes as its inputs vectors in some original space and returns the dot product of vectors in the feature space. More formally, if we have x,z∈X and a map $\phi :X\to R^{N}$, then *k*(***x***, ***z***) = 〈*ϕ*(***x***), *ϕ*(***z***)〉 is a kernel function. An important concept relating to kernel methods is the reproducing kernel Hilbert space (RKHS). Because all kernels are positive definite, there exists one or more feature spaces for which the kernel defines the inner product, without having to explicitly define such feature space. Using the Moore-Aronszajn theorem, it can be shown that for each kernel *k* on a set X, there is a unique space of functions (known as the Hilbert space) on X for which *k* is a reproducing kernel [34].

Letting *Z* be a multidimensional array of variables and *i*, *j* be two subjects, then *k*(*Z*_*i*_, *Z*_*j*_) can be used as a measure of similarity between the pair of subjects *i*, *j* since *k* (⋅, ⋅) is positive definite. This similarity measure can be can then be incorporated into a statistical inference framework to test what extent variation in *Z* between individuals can explain variation in some outcome of interest *Y*. A range of kernel functions are used in statistics, where the choice of kernel determines the function space used to approximate the relationship between two variables [46]. Within the context of this work, we will be focusing on distance-based kernels, which are denoted as

$$\begin{array}{c} K_{d}(x_{1},x_{2})=\text{exp}\;\{\frac{-d^{2}\left(x_{1},x_{2}\right)}{\rho }\}, \end{array}$$ (9)

where *d*^2^(*x*_1_, *x*_2_) is a distance function and *ρ* an unknown bandwidth or scaling parameter [34]. This will be a proper kernel for the extrinsic cluster evaluation metrics but will not be for the intrinsic metrics unless conditioned on the optimized values as computing Eq (9), where *ρ* is based on optimizing some objective function, does not guarantee that *K*_*d*_ (*x*_1_, *x*_2_) will be positive definite.

### The kernel machine model for binary outcomes

Suppose a dataset consists of *n* subjects, where for subject *i* = (1, …, *n*), *y*_*i*_ is a binary outcome taking values in {0, 1}, ***x***_*i*_ is a *q* × 1 vector of clinical covariates and ***z***_*i*_ is a *p* × 1 vector of cluster evaluation metrics. The outcome *y*_*i*_ depends on ***x***_*i*_ and ***z***_*i*_ through the following semiparametric linear model

$$\begin{array}{c} G(E(y_{i}))=x_{i}^{T}\beta +h(z_{i}), \end{array}$$ (10)

where *G*(⋅) is a link function, ***β*** is a *q* × 1 vector of regression coefficients, and *h*(***z***_*i*_) is an unknown, centered and smooth function. Here and in the sequel, we will focus on logistic regression, although the ideas and arguments can be adapted to linear regression with some modifications. Eq (10) models covariate effects parametrically and the brain connectivity metric either parametrically or non-parametrically. There are several special cases of Eq (10): when *h* (⋅) = 0, the model reduces to a standard logistic regression model. When ***x***_*i*_ = 1, the model reduces to a least squares kernel machine regression. A hypothesis test can be conducted to determine whether the multidimensional variable set ***z***_*i*_ is associated with *y*_*i*_, controlling for ***x***_*i*_, of the form

$$\begin{array}{cccc} H_{0}: & h(\cdot ) & = & 0\\ H_{A}: & h(\cdot ) & \neq & 0 \end{array}$$

[34, 46]. Assuming that *h* (⋅) lies within the function space generated by a kernel function *K* (⋅, ⋅), $h(\cdot )\in H_{k}$, *β* and *h* (⋅) can be simultaneously estimated by maximizing the penalized log likelihood function

$$\begin{array}{c} \begin{array}{cc} J[\beta ,k(\cdot )] & =\sum_{i=1}^{n}[y_{i}\;\text{ln}\;(\frac{\mu_{i}}{1-\mu_{i}})+\text{ln}\;(1-\mu_{i})]-\frac{\lambda }{2}{\left\|h\right\|}_{H_{k}}^{2}\\ & =\sum_{i=1}^{n}[Y_{i}(X_{i}^{T}\beta_{i}+h(Z_{i}))-\text{ln}\;(1+\text{exp}\;\{X_{i}^{T}\beta_{i}+h(Z_{i})\})]-\frac{\lambda }{2}{\left\|h\right\|}_{H_{k}}^{2}, \end{array} \end{array}$$ (11)

where λ is a regularization parameter that reflects the trade off between model complexity and goodness of fit [47]. At its boundaries, λ = 0 reflects a saturated model, while λ = ∞ reduces the model to a fully parametric logistic regression model. However, it should be noted that there are two unknown parameters within *J*[*β*, *h* (⋅)]: the regularization parameter λ and bandwidth parameter *ρ*. Intuitively, λ controls the magnitude of the unknown function *h* (⋅) while *ρ* controls the smoothness of *h* (⋅) [46]. The choice of *ρ* has a strong influence on the resulting estimate and choosing the data-driven, minimally optimal value of *ρ* is crucial. Using the representer theorem, which states that a solution to the penalized log likelihood function

$$\begin{array}{c} \underset{h(\cdot )\in H_{k}}{\text{min}}\;[\ell_{y}(h(x_{1}),...,h(x_{n}))+\Omega {\left\|h\right\|}_{H_{k}}^{2}] \end{array}$$ (12)

takes the form

$$\begin{array}{c} h(z_{i})=\sum_{j=1}^{n}\alpha_{j}K(z_{i},z_{j})=k_{i}^{T}\alpha \end{array}$$ (13)

[48], then the penalized log likelihood function can be rewritten as

$$\begin{array}{c} J[\beta ,\alpha ]=\sum_{i=1}^{n}[y_{i}(x_{i}^{T}\beta +k_{i}^{T}\alpha )-\text{ln}\;(1+\text{exp}\;\{x_{i}^{T}\beta +k_{i}^{T}\alpha \})]-\frac{\lambda }{2}\alpha^{T}K\alpha , \end{array}$$ (14)

where ***k***_*i*_ = {*K* (***z***_*i*_, ***z***_1_), …, *K*(***z***_*i*_, ***z***_*n*_)} and ***α*** is a *n* × 1 vector of unknown parameters. Solving for ***α*** and ***β*** gives the equations

$$\begin{array}{c} \begin{array}{cc} & \hat{\alpha }=\frac{1}{\lambda }{\left(I+\frac{K}{\lambda }\right)}^{-1}(y-X\hat{\beta })\\ & \hat{\beta }={\left[X^{T}{\left(I+\frac{K}{\lambda }\right)}^{-1}X\right]}^{-1}X^{T}{\left(I+\frac{K}{\lambda }\right)}^{-1}y \end{array} \end{array}$$ (15)

and then, plugging in $\hat{\alpha }$ from (15) into (13)

$$\begin{array}{c} \hat{h}(z)=\frac{1}{\lambda }\{K\left(z,z_{1}\right),\ldots ,K\left(z,z_{n}\right)\}{\left(I+\frac{K}{\lambda }\right)}^{-1}(y-X\hat{\beta }) \end{array}$$ (16)

[46]. However, it is possible to approach *J*[*β*, *h* (⋅)] from a generalized linear mixed models (GzLMM) perspective. As logistic regression is a special case of GzLMM, the kernel estimator within the semiparametric logistic regression model parallels the penalized quasi-likelihood function from a logistic mixed model, letting *τ* = 1/λ denote the regularization parameter and *ρ* remaining the bandwidth parameter [46]. These parameters can be treated as variance components, where *h* (⋅) ∼ *N*(0, *τ* ***K***(*ρ*)) can be treated as a subject-specific random effect and the covariance matrix ***K***(*ρ*) is an *n* × *n* kernel matrix as previously defined [47]. This means that estimating *β* and *h* (⋅) can be done by maximizing the penalized log likelihood

$$\begin{array}{c} J[\beta ,h(\cdot )]=\sum_{i=1}^{n}[y_{i}(x_{i}^{T}\beta +h(z_{i}))-\text{ln}\;(1+\text{exp}\;\{x_{i}^{T}\beta +h(z_{i})\})]-\frac{1}{2\tau }h^{T}Kh, \end{array}$$ (17)

where ***h*** = ***K****α* and *τ* = 1/λ [47]. This provides an advantage as it allows for testing of the null hypothesis *H*_0_ : *τ* = 1/λ = 0 without explicit specification of basis functions.

However, under the null hypothesis, the kernel matrix *K* disappears, which makes *ρ* a nuisance parameter that is inestimable under the null hypothesis. Davies studied the issue of a nuisance parameter disappearing under the null hypothesis [49], and proposed a score test be used, where the score statistic is treated like a nuisance parameter-indexed Gaussian process. As Eq (14) is a nonlinear function of (*α*, *β*), a Newton-Raphson algorithm needs to be implemented to maximize Eq (14) in terms of (*α*, *β*). If (*j*) is the *j*^*th*^ iteration of the algorithm, then the (*j* + 1) step solves

$$\begin{array}{c} \left[\begin{array}{cc} X^{T}D^{\left(j\right)}X & X^{T}D^{\left(j\right)}K\\ D^{\left(j\right)}X & \tau^{-1}I+D^{\left(j\right)}K \end{array}][\begin{array}{c} \beta^{\left(j+1\right)}\\ \alpha^{\left(j+1\right)} \end{array}]=[\begin{array}{c} X^{T}D^{\left(j\right)}{\tilde{y}}^{\left(j\right)}\\ D^{\left(j\right)}{\tilde{y}}^{\left(j\right)} \end{array}\right] \end{array}$$ (18)

where ${\tilde{y}}^{\left(j\right)}=X\beta^{\left(j\right)}+K\alpha^{\left(j\right)}+{\left(D^{\left(j\right)}\right)}^{-1}(y-X_{i}^{T}\beta^{\left(j\right)})$, $D^{\left(j\right)}=diag[X_{i}^{T}\beta^{\left(j\right)}-(1-X_{i}^{T}\beta^{\left(j\right)})]$, and ***h***^(*j*)^ = ***Kα***^(*j*)^. Also noting that ***β*** and *k* (⋅), which depend on *τ* and *ρ*, can be estimated using penalized quasi-likelihood under a logistic mixed model paradigm, then we can rewrite Eq (17)

$$\begin{array}{c} J(\beta (\nu ),\nu )\approx -\frac{1}{2}log\left|V\right|-\frac{1}{2}log\left|X^{T}V^{-1}X\right|-\frac{1}{2}{\left(\tilde{y}-X\beta \right)}^{T}V^{-1}(\tilde{y}-X\beta ), \end{array}$$ (19)

where *ν* = (*τ*, *ρ*) and ***V*** = ***D***^−1^ + *τ****K***. Then $\hat{\nu }$ can be solved for in the usual way [47]. However, if the derivative of Eq 13 is taken with respect to *τ*, then the score test for *H*_0_ : *τ* = 1/λ = 0 can be written as

$$\begin{array}{c} S(\rho )=\frac{Q_{\tau }(\hat{\beta_{0}},\rho )-\mu_{Q}}{\sigma_{Q}} \end{array}$$ (20)

where $Q_{\tau }(\hat{\beta_{0}},\rho )={\left(\tilde{y}-X\hat{\beta_{0}}\right)}^{T}DK\left(\rho \right)D(\tilde{y}-X\hat{\beta_{0}})={\left(\tilde{y}-\mu_{0}\right)}^{T}K(\tilde{y}-\mu_{0})$, $\hat{\beta_{0}}$ is the maximum likelihood estimate of ***β*** under the null hypothesis, $\hat{\mu_{0}}=logit^{-1}(X\hat{\beta_{0}})$, *μ*_*Q*_ = *trace*[***P***_0_***K***(*ρ*)], $\sigma_{Q}^{2}=2*trace[P_{0}K\left(\rho \right)P_{0}K\left(\rho \right)]$, ***P***_0_ = ***D***_0_ − ***D***_0_***X***(***X***^*T*^ ***D***_0_***X***)^−1^ ***X***^*T*^***D***_0_, and ***D***_0_ = *diag*[*μ*_0_ − (1 − *μ*_0_)] [47].

*S*(*ρ*) under the null hypothesis is an approximate, *ρ*-indexed Gaussian process, which requires application of Davies’ results [49] to get the upper bound for the score test’s p-value. It can be seen that large values of $Q_{\tau }(\hat{\beta_{0}},\rho )$ would result in a rejection of *H*_0_ and the upper bound of the p-value is

$$\begin{array}{c} \Phi (-M)+\frac{W\;\text{exp}\;\{-\frac{1}{2}M^{2}\}}{\sqrt{8\pi }} \end{array}$$ (21)

where Φ(⋅) is the normal cumulative distribution function, *M* is the maximum of *S*(*ρ*) over all of the searched range of *ρ*, *W* = |*S*(*ρ*_1_) − *S*(*L*)| + |*S*(*ρ*_2_) − *S*(*ρ*_1_)| + ⋯ + |*S*(*U*) − *S*(*ρ*_*m*_)|, *L* and *U* are the lower and upper bounds, respectively, of the search area for *ρ*, and *ρ*_*m*_ are the search points between *L* and *U* [47, 49]. Liu et al. suggest setting the lower and upper bounds of the *ρ* search to be $L=0.1min_{i\neq j}\sum_{l=1}^{p}{\left(z_{il}-z_{jl}\right)}^{2}$ and $U=100max_{i\neq j}\sum_{l=1}^{p}{\left(z_{il}-z_{jl}\right)}^{2}$ [47].

### The kernel machine model for continuous outcomes

The proposed semiparametric kernel machine model (10) can easily be extended to accommodate other types of outcomes. In this section, we will briefly discuss how to extend the estimation and testing procedures detailed for a binary outcome to a continuous data type.

Suppose a dataset consists of *n* subjects, where for subject *i* = (1, …, *n*), ***x***_*i*_ is a *q* × 1 vector of clinical covariates and ***x***_*i*_ is a *p* × 1 vector of cluster evaluation metrics, and *y*_*i*_ is a continuous outcome following a distribution within the exponential family density,

$$\begin{array}{c} p(x,\theta )=h(x)\;\text{exp}\;\{\eta \cdot T(X)-A(\eta )\}, \end{array}$$ (22)

where *T*(*x*) is the sufficient statistic of the distribution, or a function of the data that holds all information the data *x* provides with regards to the unknown parameter values, *η* is the natural parameter, and *A*(*η*) is the cumulant function. The mean of *y*_*i*_ satisfies *μ*_*i*_ = *E*(*y*_*i*_) = ∂*A*/∂*η* while *Var*(*y*_*i*_) = ∂^2^*A*/∂*η*^2^. The outcome *y*_*i*_ depends on ***x***_*i*_ and ***z***_*i*_ through the following generalized semiparametric linear model

$$\begin{array}{c} g(\mu_{i})=x_{i}^{T}\beta +h(z_{i})+e_{i}, \end{array}$$ (23)

where *g* (⋅) is a known monotone link function and *h* (⋅) has the same interpretation as for the logistic case. For a Gaussian-distributed outcome, *g*(*μ*) = *μ* gives us a linear kernel machine model. Thus, the regression coefficients *β* and nonparametric function *h* (⋅) can be obtained by maximizing the penalized log-likelihood function

$$\begin{array}{c} J(h,\beta )=\sum_{i=1}^{n}\ell \{y_{i},x_{i},z_{i};\beta ,h(\cdot )\}-\frac{\lambda }{2}{\left\|h\right\|}_{H_{k}}^{2}, \end{array}$$ (24)

where *ℓ* (⋅) = log (*p*), *p* is the density given in Eq (22), and λ is a tuning parameter. Using the kernel expression of *h* (⋅) specified in Eq (13), we can write

$$\begin{array}{c} g(\mu_{i})=x_{i}^{T}\beta +k_{i}^{T}\alpha , \end{array}$$ (25)

and the penalized likelihood can be rewritten as

$$\begin{array}{c} J(\beta ,\alpha )=\sum_{i=1}^{n}\ell (y_{i},x_{1},z_{1};\beta ,\alpha )-\frac{1}{2}\lambda \alpha^{T}K\alpha , \end{array}$$ (26)

where ***K*** is a *n* × *n* matrix whose *i*, *j*^*th*^ element is *K*(***z***_*i*_, ***z***_*j*_). From here, the procedure is essentially the same as for the binary outcome case, with *μ*_*i*_ specified under 10 and ***D*** = *diag*{*var*(*y*_*i*_)} under 22. The score test has a straightforward alteration with the only change occurring in ***D***, where the variance needs to be replaced with the appropriate variance function for *y*_*i*_.

### Simulated datasets

**Fig 1:**
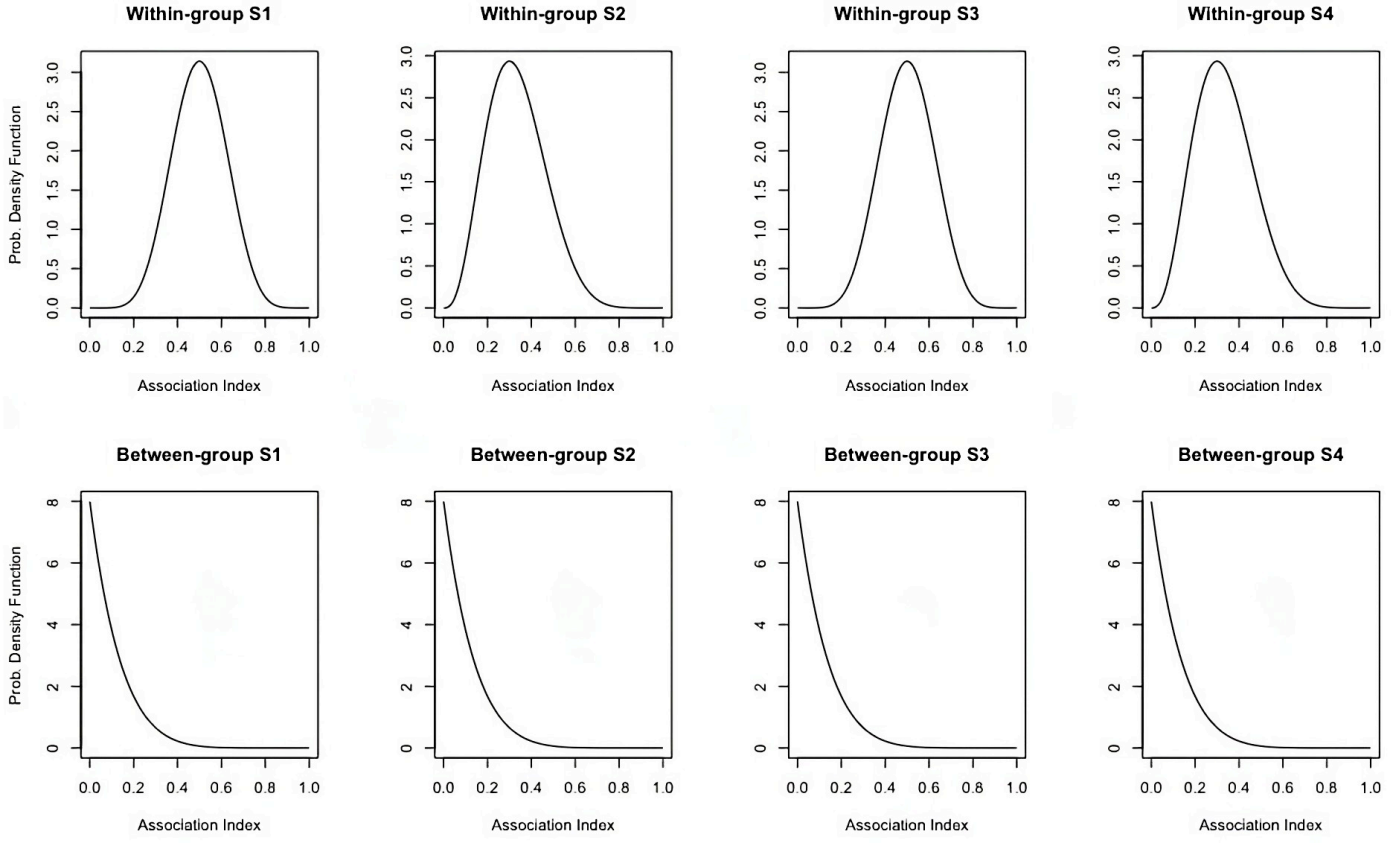
Examples of between- and within-community Beta distributions. Four examples of Beta distributions simulated to reflect the distribution function from which either within-community (top panel) or between-community (bottom panel) edge weights that would be sampled in the simulated network.

To understand the statistical properties of the kernel machine tests using either extrinsic or intrinsic cluster evaluation metrics, simulated networks with community structure were constructed. In order to simulate similar community structures amongst a group of networks with a corresponding level of inter-subject variability, Beta distributions were utilized. As functional connectivity within real brain imaging data is typically assessed using some form of correlation and then bounded to be non-negative, the support of the Beta distribution, [0, 1], fits nicely within these restrictions. Two different Beta distributions were simulated for each group of networks: one representing between-community edges and one representing within-community edges. Fig 1 provides four examples of beta distributions simulated to reflect either between-community (bottom panel) or within-community (top panel) edge weights that would be sampled for a simulated group of networks. Well-defined community structure, as well as ill-defined community structure, can be simulated through the shape parameters (*α* and *β*).

**Fig 2:**
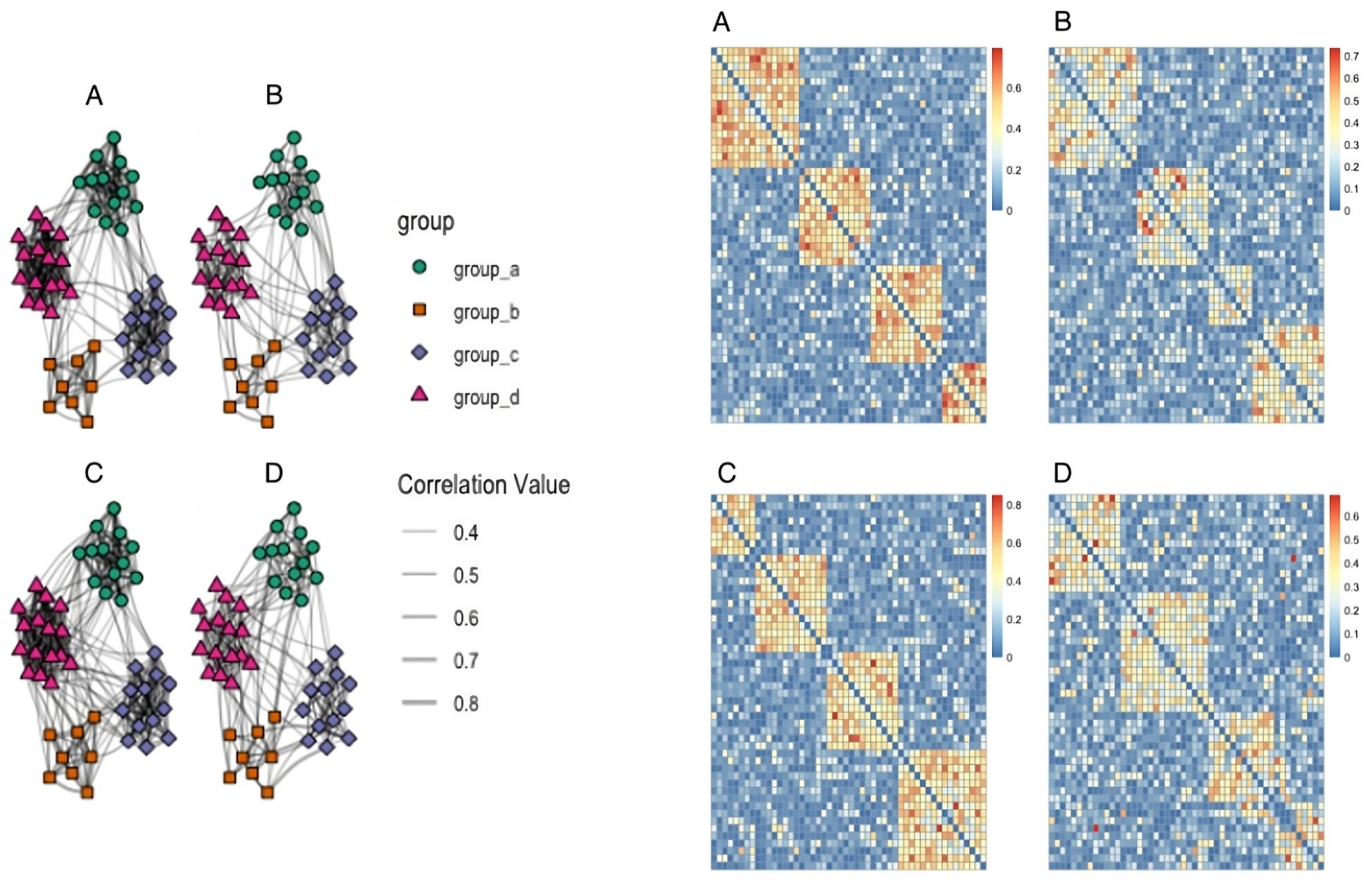
Examples of four simulated networks and adjacency matrices. Four examples of simulated networks of 50 nodes each and four communities. For all simulated networks, the between-group edge weights were sampled from *Beta*(1, 8). For panels A and C, the within-group edge weights were sampled from *Beta*(8, 8) while for panels B and D they were sampled from *Beta*(4, 8).

To create the simulated networks, a data structure is first created with node labels and community memberships of these nodes is randomly assigned. “Edge weights” are assigned to all dyadic relationships. If the dyad’s nodes belong the same community, the within-community beta distribution is sampled; if the dyad’s nodes belong to different communities, the between-community beta distribution is sampled. Fig 2 provides an example of four simulated networks, all with 50 nodes and four communities. For all four simulated networks, the between-group edge weights were sampled from *Beta*(1, 8). For panel A and C, the within-group edge weights were sampled from *Beta*(8, 8) while for panel B and D they were sampled from *Beta*(4, 8). As modeled in the figure, panel A and C are very similar, with C having slightly higher within-community edge weights. Panels B and D have comparatively weaker within-group edge weights compared to their panel A and C counterparts.

Extensions of these simulations allow for perturbation of the community assignment for nodes to create additional variability within networks. A “master” community assignment is created and then, for each network, a pre-specified proportion of nodes are randomly selected and reassigned to a new community. From there, the edge weights are assigned to the dyads in the same manner as previously described.

### ABIDE dataset

De-identified and pre-processed data from the Autism Brain Imaging Data Exchange (ABIDE) [50] was provided as part of the International Neuroimaging Datasharing Initiative (INDI) consortium’s work with the Pre-Processed Connectomes Project (PCP) (http://preprocessed-connectomes-project.org/abide/index.html). A total of 1112 datasets, 539 from individuals with Autism Spectrum Disorder (ASD) and 573 typical controls, were aggregated from 16 international imaging sites. For each subject included in ABIDE, a high resolution, 1*mm*^3^ isotropic T1-weighted scan was obtained using a MPRAGE sequence and a resting-state functional MRI (fMRI) was obtained using a BOLD contrast.

The Configurable Pipeline for the Analysis of the Connectomes (C-PAC) pipeline was chosen for the functional pre-processing. The fMRI data was slice time corrected, motion corrected, and the voxel intensity was normalized. Nuisance signal removal was performed using 24 motion parameters, CompCor with five components [51] and low-frequency drifts (linear and quadratic trends), without any global signal regression. Functional data was band-pass filtered (0.01-0.1Hz) and spatially registered using a nonlinear technique to the MNI152 template space. The mean time series for regions of interest were extracted for each subject. The Craddock 200 functional parcellation atlas of the brain [52] was used to reduce the feature vector size. This atlas was generated using a data-driven parcellation of the whole brain into spatially close regions of homogeneous functional activity, resulting in 200 regions. Labels were generated for each of the resulting ROIs from their overlap with the Automated Anatomical Labelling, Eickhoff-Ziles, Harvard-Oxford, and Talaraich and Tournoux atlases. Phenotypic information, including age at time of scan, sex, handedness, body mass index (BMI), and an array of neurocognitive testing results were also made available for all subjects.

A stratified random sample was taken from the larger ABIDE pre-processed database. Because every subject in the pre-processed ABIDE database was registered to the same MNI152 template, subjects under 18 years of age or over 45 years of age were excluded due to potential biasing effects during the registration step. The remaining pool of potential subjects were then categorized into one of three buckets: 18-24, 25-34, or 35-45 years of age. This age categorization and the diagnosis group (diagnosed ASD or control) were used in the stratified randomization. Functional connectivity was assessed using partial correlation coefficients. This coefficient is an index of the functional connection of two regions of the brain, along the [−1, 1] range. Negative correlations were zeroed out due their inability to be included in the vast majority of community detection algorithms. Communities were determined using two different algorithms: the Louvain method first proposed by [53] and the spinglass algorithm first proposed by [40]. This was done to understand the robustness of the kernel machine model to choice of community detection algorithm. Resulting community structures as well as the optimized modularity (for the Louvain algorithm) or Hamiltonian (for the spinglass algorithm) values were extracted for each subject’s run through the community detection method.

## Results

### Simulated datasets

To analyze the robustness of the logistic kernel machine model as previously detailed, several simulations were conducted using the simulated network approach previously described. These simulations were split between the number of groups of functional connectivity matrices that were generated. A simulation under a one generation process presumes that the null hypothesis is true (i.e., all subjects come from the same underlying population) while a two generation process presumes that the null hypothesis is false and subjects come from two distinct populations. To simplify the analyses for these simulations, no covariates were generated.

A series of simulation studies were conducted to evaluate the performance of the kernel machine model under the hypothesis test of *H*_0_ : *h* (⋅) = 0 versus *H*_*A*_ : *h* (⋅) ≠ 0. As there is no closed-form solution solution for the test statistic’s accompanying p-value, power and Type I error were determined empirically. For each of the 1000 power simulation iterations, 100 different networks were produced, with a 45/55 split between “cases” and “controls.” For the cases, the between-community edge weights were sampled from *Beta*(1.5, 8) and the within-community edge weights were sampled from *Beta*(7, 8). For the controls, the between- and within-community edge weights were sampled from *Beta*(1.5, 8) and *Beta*(8, 8), respectively. Each network contained 50 nodes and three communities, with a community assignment perturbation probability set at 0.1. Bounds of the *ρ* search were set based on the suggestion from [47]. An indicator function was used to determine whether each simulation’s resulting p-value was greater than *α* = 0.05, with the empirical power calculated as the proportion of iterations with a p-value<0.05. The Louvain and spinglass algorithms were each used to determine community assignment for the simulated datasets. Power simulations were conducted for each of the four different cluster evaluation metrics: optimized modularity/Hamiltonian value, purity, NMI, and ARI.

Similarly, for each of the 1000 Type I error simulation iterations, 100 different networks were produced, all with the same between-community edge weights sampled from *Beta*(1.5, 8) and within-community edge weight sampled from *Beta*(8, 8). Each network contained 50 nodes and three communities, with a community assignment perturbation probability set at 0.1. Networks were randomly assigned to either a “case” or “control” group with a 45/55 split. Bounds of the *ρ* search were again set based on the suggestion from [47]. An indicator function was used to determine whether each simulation’s resulting p-value was less than *α* = 0.05, with the empirical Type I error calculated as the proportion of iteration with a p-value<0.05. The Louvain and spinglass algorithms were each used to determine community assignment and Type I error simulations were conducted for the same four cluster evaluation metrics.

Table 1, below, details the power and Type I error results for all of the cluster evaluation metric simulations. As can be seen, both the intrinsic cluster evaluation metrics (optimized modularity/Hamiltonian value) and the three extrinsic metrics (NMI, ARI, and purity) had comparable power and Type I errors for the nonparametric kernel logistic regression.

**Table 1:** Logistic kernel: Power and Type I error for cluster evaluation metric simulations.

	Louvain		Spinglass	
	Power	Type I Error	Power	Type I Error
Optimized Algorithmic Value	0.940 (SE: 0.008)	0.038 (SE: 0.006)	0.984 (SE: 0.004)	0.040 (SE: 0.006)
Normalized Mutual Information (NMI)	0.990 (SE: 0.003)	0.030 (SE: 0.005)	0.990 (SE: 0.003)	0.012 (SE: 0.003)
Adjusted Rand Index (ARI)	0.986 (SE: 0.004)	0.018 (SE: 0.004)	0.988 (SE: 0.003)	0.018 (SE: 0.004)
Purity	0.976 (SE: 0.005)	0.024 (SE: 0.005)	0.981 (SE: 0.004)	0.023 (SE: 0.005)

Power and Type I error simulations were also conducted for the linear kernel machine method to evaluate its performance under the hypothesis test of *H*_0_ : *h* (⋅) = 0 versus *H*_*A*_ : *h* (⋅) ≠ 0. Again, simulations were used as there is no closed form solution for the test statistic’s p-value. For each of the 1000 power simulation iterations, 100 different networks were produced, with a 45/55 split between “cases” and “controls.” For the cases, the between-community edge weights were sampled from *Beta*(1.5, 8) and the within-community edge weights were sampled from *Beta*(7, 8). For the controls, the between- and within-community edge weights were sampled from *Beta*(1.5, 8) and *Beta*(8, 8), respectively. Each network contained 50 nodes and three communities, with a community assignment perturbation probability set at 0.1. Two covariates—one continuous and one binary—were randomly simulated using a *N*(*μ* = 10, *σ* = 5) and *Binom*(*n* = 100, *p* = 0.25) distribution, respectively. The outcome variable was distributed under the following statistical model: $y_{i}=\beta_{0}+\beta_{1}*(T_{group_{i}=1})+\epsilon_{i}$, where *β*_0_ = 5, *β*_1_ = −4, *ϵ* ∼ *N*(*μ* = 0, *σ* = 2). Bounds of the *ρ* search were set based on the suggestion from [47]. An indicator function was used to determine whether each simulation’s resulting p-value was greater than *α* = 0.05, with the empirical power calculated as the proportion of iterations with a p-value < 0.05 These power simulations were conducted for the four different cluster evaluation metrics: optimized modulairty/Hamiltonian value, purity, NMI, and ARI.

Similarly, for each of the 1000 Type I error simulation iterations, 100 different networks were produced, all with the same between-community edge weights sampled from *Beta*(1.5, 8) and within-community edge weight sampled from *Beta*(8, 8). Each network contained 50 nodes and three communities, with a community assignment perturbation probability set at 0.1. Networks were randomly assigned to either a “case” or “control” group with a 45/55 split. Two covariates—one continuous and one binary—were randomly simulated using a *N*(*μ* = 10, *σ* = 5) and *Binom*(*n* = 100, *p* = 0.25) distribution, respectively. The outcome variable was similarly randomly generated under a *N*(*μ* = 100, *σ* = 15) distribution. Bounds of the *ρ* search were again set based on the suggestion from [47]. An indicator function was used to determine whether each simulation’s resulting p-value was less than *α* = 0.05, with the empirical Type I error calculated as the proportion of iterations with a p-value<0.05. These Type I error simulations were conducted for the four different cluster evaluation metrics: optimized modularity/Hamiltonian value, purity, NMI, and ARI.

Table 2, below, details the power and Type I error results for all of the cluster evaluation metric simulations. As can be seen, both the intrinsic cluster evaluation metric (Hamiltonian value) and the three extrinsic metrics (NMI, ARI, and purity) had comparable power and Type I errors for the semiparametric linear kernel regression, and had comparable test characteristics to the semiparametric logistic kernel regression.

**Table 2:** Linear kernel: Power and Type I error for cluster evaluation metric simulations.

	Louvain		Spinglass	
	Power	Type I Error	Power	Type I Error
Optimized Algorithmic Value	0.884 (SE: 0.010)	0.060 (SE: 0.008)	0.978 (SE: 0.005)	0.042 (SE: 0.006)
Normalized Mutual Information (NMI)	0.990 (SE: 0.003)	0.012 (SE: 0.003)	0.987 (SE: 0.004)	0.010 (SE: 0.003)
Adjusted Rand Index (ARI)	0.985 (SE: 0.004)	0.006 (SE: 0.002)	0.983 (SE: 0.004)	0.020 (SE: 0.004)
Purity	0.988 (SE: 0.003)	0.018 (SE: 0.004)	0.979 (SE: 0.005)	0.012 (SE: 0.003)

For both the logistic and linear kernel machine methods, the extrinsic cluster evaluation metrics (NMI, ARI, and purity) all displayed higher power and lower Type I error rates as compared to the intrinsic metric. A higher level of inter-subject variability exists for the optimized modularity/Hamiltonian value, due to not only the potential differences in the tolerance stopping criteria of the communitiy detection algorithm implemented but also the influence of the distribution of edge weights within and between communities. These effects may be more pronounced for the Louvain community detection algorithm, which saw a larger difference in power and Type I error for both the logistic and linear kernel machine methods.

### ABIDE dataset

Table 3 provides a summary of the phenotypic information on the subsampled ABIDE dataset, both overall and by diagnosis group. Initially, the subsample included 85 subjects, but two had to be excluded due to substantial missingness issues in their fMRI time series data, resulting in the analytic sample size of *n* = 83. As can be seen, the male/female split is quite skewed, but this is not surprising given ASD is identified in females at a substantially lower rate than males, with many studies reporting an approximate 4:1 male to female ratio [54]. Handedness was more evenly distributed for those with ASD as compared to controls. Full-scale IQ scores were equivalent, as was the distribution of subjects to the 10 sites in the subsample. It should be noted that in the larger ABIDE sample, there were a total of 20 sites. However, 10 of these sites only imaged pediatric subjects, excluding them from this subsample.

**Table 3:** ABIDE dataset subject demographics.

	Overall (n = 83)	Controls (n = 44)	ASD (n = 39)
Age in years, mean (SD)	26.50 (6.63)	27.12 (7.01)	25.81 (6.18)
Sex, n (%)			
Male	75 (90.4%)	37 (84.1%)	38 (97.4%)
Female	8 (9.6%)	7 (15.9%)	1 (2.6%)
Handedness, n(%)			
Ambidextrous	2 (4.1%)	1 (3.6%)	1 (4.8%)
Left	3 (6.1%)	0 (0.0%)	3 (14.3%)
Right	34 (87.8%)	26 (92.9%)	17 (81.0%)
NA	1 (2.0%)	1 (3.6%)	0 (0.0%)
Site, n(%)			
California Inst of Tech	10 (12.0%)	6 (13.6%)	4 (10.3%)
Carnegie Mellon Univ	7 (8.4%)	5 (11.4%)	2 (5.1%)
Univ of Leuven, Sample 1	7 (8.4%)	3 (6.8%)	4 (10.3%)
Ludwig Maximilians Univ Munich	8 (9.6%)	4 (9.1%)	4 (10.3%)
New York Univ	11 (13.3%)	7 (15.9%)	4 (10.3%)
Olin Inst of Living at Hartford Hosp	4 (4.8%)	1 (2.3%)	3 (7.7%)
Univ of Pittsburgh	7 (8.4%)	5 (11.4%)	2 (5.1%)
Social Brain Lab, Netherlands	10 (12.0%)	4 (9.1%)	6 (15.4%)
Trinity Centre for Health Sciences	5 (6.0%)	4 (9.1%)	1 (2.6%)
Univ of Utah	14 (16.9%)	5 (11.4%)	9 (23.1%)

Quality checks were performed for the fMRI times series data, which included looking at missingness of time series data within regions of interest (ROIs), summary statistics, and time series plots. Three functionally and anatomically diverse ROIs (thalamus, frontal orbital cortex, and middle occipital gyrus) were plotted to check for range of time series values and overall trends. During this quality assurance process, four ROIs had missing time series data for at least one subject:

- Right temporal pole (missing for one subject)
- left temporal pole (missing for eight subjects)
- left cerebellum (missing for one subject)
- right temporal fusiform cortex, anterior (missing for one subject)

All four of these ROIs are located in the most inferior portion of the brain, where the signal-to-noise ratio is especially poor due to its proximity to the spinal column, skeleton, and sinus cavity. Depending on the field of view of the scan, the inferior part of the brain can be cut off. As the assumption for all cluster evaluation metrics, whether intrinsic or extrinsic, is that the input (the network) is the same, these four ROIs were dropped from all subjects’ fMRI datasets. The final analytic fMRI dataset was, therefore, 196 ROIs in size.

**Fig 3:**
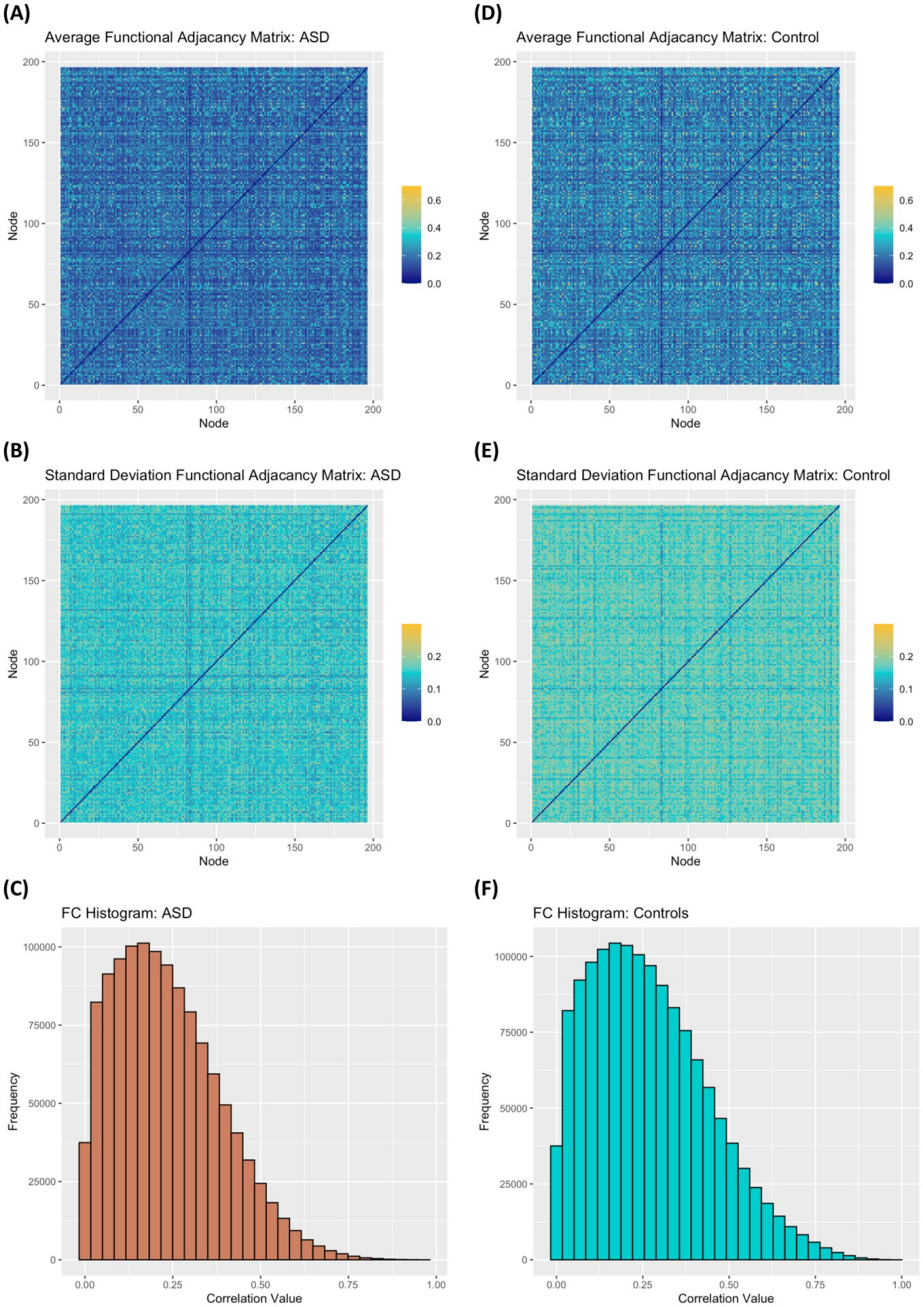
Distribution of cross correlations in functional connectivity in ABIDE sample. For subjects with ASD: *(A)* Average functional connectivity matrix; *(B)* Standard deviation of the functional connectivity matrix; *(C)* Cross correlation distribution of functional connectivity. For control subjects: *(D)* Average functional connectivity matrix; *(E)* Standard deviation of the functional connectivity matrix; *(F)* Cross correlation distribution of functional connectivity.

Fig 3 provides a visual summary of the functional connectivity for subjects with ASD versus control subjects. As can be seen, average and standard deviation functional connectivity matrices for the two groups look overall very similar, though the histograms show a slightly wider range of possible values, with a higher average correlation value for controls (${\bar{x}}_{control}=0.22$) compared to those with ASD (${\bar{x}}_{ASD}=0.19$) and a higher standard deviation, *s*_*control*_ = 0.17 and *s*_*ASD*_ = 0.15, respectively.

Neither community detection method required the pre-specification of the number of communities. The Louvain method is agglomerative, where each node starts as its own community and the method iteratively merges communities if doing so increases the partition’s modularity. The process stops if no such merge is possible. Therefore, the number of communites does not need to be specified a priori but rather is determined algorithmically. The spinglass algorithm does require a pre-specification of the maximum number of possible communities (*max*(*n*_*c*_)) but the optimal solution can include empty communities (or spin states). The choice of *max*(*n*_*c*_) is a balance between finding the optimal, data-driven partition and computation time. The preferable number of communities was found through an exploratory analysis, whereby a random subset of 10 subjects from the analytic sample were selected and the spinglass algorithm was run, varying *max*(*n*_*c*_) from 3 to 10 communities to determine the consensus number of communities being used by the algorithm across subjects. We recommend such exploratory analysis for any real dataset when using the spinglass algorithm for community detection.

For the binary outcome of diagnosis group (ASD versus control), three different regression models were tested. First, a fully nonparametric logistic model was considered, which did not include any covariates to be parametrically estimated:

$$\begin{array}{c} logit[\text{Pr}\;(y=1)]=h(Z), \end{array}$$ (27)

where ***Z*** is the multidimensional distance matrix representing one of the four cluster evaluation metrics: Hamiltonian value, NMI, ARI, or purity. Next, a simple semiparametric logistic model was considered, where the only covariate parametrically estimated was site:

$$\begin{array}{c} logit[\text{Pr}\;(y=1)]=\beta_{0}+\beta_{1}*(site)+h(Z). \end{array}$$ (28)

Previous studies have shown considerable variability in fMRI measurements due to different scanner manufacturers, acquisition protocols, and even climate effects [55, 56]. Therefore, site was included in the model to control for this potential source of confounding between the cluster evaluation metrics and diagnostic group. Finally, a fuller semiparametric model was fit to control for both site-specific effects and subject-level effects of age and sex:

$$\begin{array}{c} logit[\text{Pr}\;(y=1)]=\beta_{0}+\beta_{1}*(site)+\beta_{2}*(age)+\beta_{3}*(sex)+h(Z). \end{array}$$ (29)

Table 4 details the results of the score tests for the logistic kernel score tests under the hull hypothesis of *H*_0_ : *h* (⋅) = 0 for all three regression models and the four different cluster evaluation metrics using both the Louvain and spinglass approaches for community detection. In all cases, we failed to reject the null hypothesis. Interestingly, inclusion of potential confounders affected the p-values for the intrinsic and extrinsic metrics in opposing directions when using the spinglass community detection algorithm while p-values for all metrics were affected in a simlar manner when using the Louvain approach for community detection.

**Table 4:** P-values from analysis of ABIDE dataset for binary outcome of diagnostic group.

**(a)** Louvain algorithm				
Regression	Modularity	NMI	ARI	Purity
Non-parametric	*p* = 0.643	*p* = 0.398	*p* = 0.420	*p* = 0.462
Semiparametric: Site only	*p* = 0.325	*p* = 0.262	*p* = 0.265	*p* = 0.309
Semiparametric: Site, Age, Sex	*p* = 0.349	*p* = 0.218	*p* = 0.220	*p* = 0.230
**(b)** Spinglass algorithm				
Regression	Hamiltonian	NMI	ARI	Purity
Non-parametric	*p* = 0.126	*p* = 0.506	*p* = 0.492	*p* = 0.486
Semiparametric: Site only	*p* = 0.371	*p* = 0.287	*p* = 0.320	*p* = 0.328
Semiparametric: Site, Age, Sex	*p* = 0.317	*p* = 0.220	*p* = 0.273	*p* = 0.274

Similarly, the three regression models specified for the binary outcome of diagnostic group, Eqs (27)–(29), were fit for the continuous outcome of verbal IQ score. Intelligence tests, while generally controversial, are an important part of the diagnostic assessment for individuals with suspected ASD. Previous studies have shown that while IQ tests appear to be equivalent and interchangeable in control subjects, this may not be the case for individuals with ASD [57, 58]. This is especially the case with verbal IQ [57]. Within the ABIDE dataset, full-scale intelligence quotient (FIQ), verbal IQ (VIQ), and performance IQ (PIQ) were derived from the Wechsler Abbreviated Scales of Intelligence (WASI), which has a scale from [50, 160]. However, verbal IQ scores were missing for *n* = 19 subjects in the analytic dataset and had to be dropped. This subset, therefore, included a total of 64 subjects, 29 with ASD and 35 controls.

Table 5 details the results of the score tests for the linear kernel score tests under the hull hypothesis of *H*_0_ : *h* (⋅) = 0 for all three regression models and the four different cluster evaluation metrics. As can be seen, using the spinglass algorithm, several cluster evaluation metrics under the semiparametric paradigms were found to reject the null hypothesis at the *α* = 0.05 significance level; all of these significant p-values came from the extrinsic cluster evaluation metrics. While no such statistical significance was found using the intrinsic metric for either the Louvain or spinglass algorithms, this could be due to a variety of factors. As previously detailed, there is a higher level of inter-subject variability for the optimized modularity/Hamiltonian value, due to not only the potential differences in the tolerance stopping criteria of the algorithms but also the influence of the distribution of edge weights within and between communities.

**Table 5:** P-values from analysis of ABIDE dataset for continuous outcome of VIQ.

**(a)** Louvain algorithm				
Regression	Modularity	NMI	ARI	Purity
Non-parametric	*p* = 0.441	*p* = 0.434	*p* = 0.449	*p* = 0.473
Semiparametric: Site only	*p* = 0.091	*p* = 0.133	*p* = 0.145	*p* = 0.169
Semiparametric: Site, Age, Sex	*p* = 0.064	*p* = 0.066	*p* = 0.074	*p* = 0.084
**(b)** Spinglass algorithm				
Regression	Hamiltonian	NMI	ARI	Purity
Non-parametric	*p* = 0.689	*p* = 0.213	*p* = 0.300	*p* = 0.238
Semiparametric: Site only	*p* = 0.351	*p* = 0.048	*p* = 0.051	*p* = 0.019
Semiparametric: Site, Age, Sex	*p* = 0.227	*p* = 0.020	*p* = 0.022	*p* = 0.005

As a comparison, each subject’s optimized algorithmic value was included in conventional regression frameworks for both the binary outcome of diagnostic group and the continuous outcome of verbal IQ score. Table 6 provides the p-values for the regression coefficient associated with the optimized value cluster evaluation metric. In all cases, this measure was not found to be significantly associated with either the binary outcome of diagnostic group or continuous outcome of verbal IQ. Because of the nature of all the external cluster evaluation metrics—normalized mutual information, adjusted Rand Index, and purity—their incorporation into a conventional regression framework was not possible. However, given the comparable empirical power and Type I error rates for all four cluster evaluation metrics for both the Louvain and spinglass community detection algorithms, we believe the conventional regression frameworks in Table 6 provide a sufficient approach comparison to the kernel machine methods.

**Table 6:** Analysis of ABIDE dataset: Conventional regression frameworks for optimized algorithmic value.

	Louvain		Spinglass	
Diagnostic Group				
	Modularity value only	*p* = 0.804	Hamiltonian value only	*p* = 0.095
	Modularity, site	*p* = 0.473	Hamiltonian, site	*p* = 0.458
	Modularity, site, age, sex	*p* = 0.309	Hamiltonian, site, age, sex	*p* = 0.402
Verbal IQ				
	Modularity value only	*p* = 0.351	Hamiltonian value only	*p* = 0.882
	Modularity, site	*p* = 0.167	Hamiltonian, site	*p* = 0.989
	Modularity, site, age, sex	*p* = 0.581	Hamiltonian, site, age, sex	*p* = 0.601

## Discussion

In this work, we proposed a kernel machine methodolgical approach for testing the association between cluster evaluation metrics and an outcome of interest, whether binary or continuous. Additional covariates can be modeled parametrically while cluster evaluation metrics are measured nonparametrically, allowing for its relationship to the outcome to be modeled without making any assumption as to the parametric form of its association. Our approach has the flexibility to incorporate not only extrinsic or intrinsic cluster evaluation metrics, but more general distance-based metrics, allowing for broad utilization of this approach. With our application to brain connectivity, by incorporating these notions of similarity into a kernel distance function, the high-dimensional feature space of brain networks, defined on input pairs, can be generalized to non-linear spaces; this allows for a wider class of distance-based algorithms, rather than the restrictive squared distance, to be representative of the similarity between two network community structures. The semiparametric form of these kernel machine methods also allow for potential confounders to be controlled for within a parametric framework, allowing for ease of parameter estimate interpretation, should they be desired. Our simulation studies showed the linear and logistic kernel machine regressions both performed well using either extrinsic or intrinsic cluster evaluation metrics, regardless of the underlying community detection algorithm implemented. Further, despite the high degree of flexibility, our proposed methodology is also computationally efficient. While we did not find significant associations between the cluster evaluation metrics and the outcomes of interest in the ABIDE dataset application, we do not see this as a limitation to our proposed method. Our kernel machine approach serves as a proof of concept for the incorporation of the results of community detection algorithms into questions of association with outcomes of interest, whether continuous or binary, without assuming the functional form of the association. Though we focused on brain functional connectivity in this study, the kernel machine approach can be more broadly applied to any type of network analysis (e.g., social networks, metabolomics, genomics, etc.).

Several methodological considerations are relevant for this work. First, while the kernel machine methods have been shown to have high power and low Type I error rate, they can only be applied to a single layer of communities at a time. When applied to the pre-processed ABIDE subset, no significant associations were found between any of the cluster evaluation metrics and the binary outcome of diagnostic group. However, the linear kernel method showed several significant associations between the extrinsic cluster evaluation metrics and verbal IQ score when using the spinglass algorithm. The simulated results combined with the real data analysis provide evidence of the robustness of this method within a single layer of communities, thus showing the potential utility of multilayer extensions to community detection algorithms. As well, use of partial correlation as the metric for functional connectivity in the ABIDE dataset could be creating interference in the robustness of the connectivity networks themselves. The suppression of the negative partial correlation values could also be masking important information about subjects’ functional connectivity; while there exist many algorithms to discover the community structure of networks, there is a limited selection that can account for negative links. As such, we focused on the more widely used community detection algorithms, which do not allow for negative network links, to provide a proof-of-concept and allow for exploration of extensions in future research. Alternatives such as wavelet coherence could be explored, though further research into the frequency band of interest, and whether that differs when applying to subjects with neurodivergence or neuropsychological diseases, needs to to be thoroughly investigated. Finally, choice of community detection algorithm on the network data could have a downstream impact on the results of the kernel regressions. For this work, we decided to focus on two community detection algorithms that showed robust performance by Yang, Algesheir, and Tessone [22]: the Louvain (or multilevel) and spinglass algorithms. On a set of Lancichinetti, Fortunato & Radicchi (LFR) benchmark graphs, the accuracy and computation time for the Louvain and spinglass algorithms was found to be comparable for increasing levels of the mixing parameter and network size (number of nodes). As such, we chose to focus on community detection algorithms that showed a high level of accuracy across a variety of LFR benchmark scenarios but differed in their discovery approaches (modularity maximization versus ground state energy). This was done to control for variability due to the community detection algorithm and focus on variability due to either cluster evaluation metric and/or the kernel machine test of association itself. A comparison of community detection algorithm performance is outside the scope of this work, but has been previously examined in depth by [59], [22], and [23].

Potential future directions of this work could extend the semiparametric kernel machine methods to account for nested hierarchical structures, which may be a more accurate representation of brain connectivity’s integrated and segregated features. This could be done in one of two ways: (1) through the incorporation of random effects or (2) through a multiple comparisons correction to the score test’s p-value. [60] derived a distance-based kernel association test for generalized linear mixed models for use in microbiome data. This derivation could be leveraged for our purposes to control for the nested hierarchical structure of communities in brain connectivity data. Similarly, [61] presented a statistical method to test for the association between a response variable and a fixed tree structure across all levels of the hierarchy while controlling for the overall false positive error rate. While their approach is based on tree structures, rather than nested hierarchical clusterings, their derivation of a multiscale statistic that includes a penalty term for multiple comparisons could be modified for use in the kernel score test of association.

Another future direction of this work would be to investigate other methods for incorporating summaries of the community structure—or the community structure itself—into the kernel machine framework. While we explored both extrinsic and intrinsic cluster evaluation metrics in this work, there is active work in the literature on ways to assess a community detection algorithm’s performance and comparing communities across networks. Understanding how advances in the literature affect the kernel machine methods generally and in the context of brain connectivity would be an important step in the argument in favor of kernel methods.
